# Long-term effectiveness of carglumic acid in patients with propionic acidemia (PA) and methylmalonic acidemia (MMA): a randomized clinical trial

**DOI:** 10.1186/s13023-021-02032-8

**Published:** 2021-10-11

**Authors:** Majid Alfadhel, Marwan Nashabat, Mohammed Saleh, Mohammed Elamin, Ahmed Alfares, Ali Al Othaim, Muhammad Umair, Hind Ahmed, Faroug Ababneh, Fuad Al Mutairi, Wafaa Eyaid, Abdulrahman Alswaid, Lina Alohali, Eissa Faqeih, Mohammed Almannai, Majed Aljeraisy, Bayan Albdah, Mohamed A. Hussein, Zuhair Rahbeeni, Ali Alasmari

**Affiliations:** 1 Genetics and Precision Medicine department (GPM), King Abdullah Specialized Children’s Hospital (KASCH), King Abdullah International Medical Research Center, King Saud Bin Abdulaziz University for Health Sciences (KSAUHS), King Abdulaziz Medical City, Ministry of National Guard Health Affairs (MNG-HA), Riyadh, Saudi Arabia; 2 Medical Genetics Section, King Fahad Medical City, Children’s Hospital, Riyadh, Kingdom of Saudi Arabia; 3 Department of Pediatrics, College of Medicine, Qassim University, Buraidah, Kingdom of Saudi Arabia; 4 Department of Pathology, King Abdullah International Medical Research Centre, King Saud bin Abdulaziz University for Health Science, King Abdulaziz Medical City, Ministry of National Guard-Health Affairs (NGHA), Riyadh, Kingdom of Saudi Arabia; 5 Medical Genomics Research Department, King Abdullah International Medical Research Center (KAIMRC), King Saud bin Abdulaziz University for Health Sciences, King AbdulAziz Medical City, Ministry of National Guard Health Affairs, Riyadh, Kingdom of Saudi Arabia; 6 King Abdullah International Medical Research Centre, College of Pharmacy, King Saud bin Abdulaziz University for Health Science, King Abdulaziz Medical City, Ministry of National Guard-Health Affairs, Riyadh, Kingdom of Saudi Arabia; 7 Department Biostatistics and Bioinformatics, King Abdullah International Medical Research Centre, King Saud bin Abdulaziz University for Health Science, Ministry of National Guard-Health Affairs, Riyadh, Kingdom of Saudi Arabia; 8 Department of Medical Genetics, King Faisal Specialist Hospital and Research Center, Riyadh, Kingdom of Saudi Arabia

**Keywords:** Carglumic acid, Hyperammonemia, Methylmalonic academia, Organic academia, Propionic acidemia

## Abstract

**Background:**

Propionic acidemia (PA) and methylmalonic acidemia (MMA) are rare, autosomal recessive inborn errors of metabolism that require life-long medical treatment. The trial aimed to evaluate the effectiveness of the administration of carglumic acid with the standard treatment compared to the standard treatment alone in the management of these organic acidemias.

**Methods:**

The study was a prospective, multicenter, randomized, parallel-group, open-label, controlled clinical trial. Patients aged ≤ 15 years with confirmed PA and MMA were included in the study. Patients were followed up for two years. The primary outcome was the number of emergency room (ER) admissions because of hyperammonemia. Secondary outcomes included plasma ammonia levels over time, time to the first episode of hyperammonemia, biomarkers, and differences in the duration of hospital stay.

**Results:**

Thirty-eight patients were included in the study. On the primary efficacy endpoint, a mean of 6.31 ER admissions was observed for the carglumic acid arm, compared with 12.76 for standard treatment, with a significant difference between the groups (p = 0.0095). Of the secondary outcomes, the only significant differences were in glycine and free carnitine levels.

**Conclusion:**

Using carglumic acid in addition to standard treatment over the long term significantly reduces the number of ER admissions because of hyperammonemia in patients with PA and MMA.

**Supplementary Information:**

The online version contains supplementary material available at 10.1186/s13023-021-02032-8.

## Background

Organic acidemias, such as propionic acidemia (PA, OMIM #606054) and methylmalonic acidemia (MMA, OMIM #251000), are rare, autosomal recessive inborn errors of metabolism. Patients usually present symptoms such as acidosis, recurrent vomiting, and poor feeding in the neonatal period. If left untreated, they progress to encephalopathy, coma, or even death. Patients tend to exhibit developmental delay, secondary hyperammonemia, cardiomyopathy, bone marrow suppression, and metabolic stroke. The prevalence of MMA ranges from 1:50,000 to 1:100,000 newborns [1], while that of PA may range from 1:2000 in the Middle East to 1:250,000 in Europe [2].

PA is caused by pathogenic variants of the *PCCA* (OMIM #232050) or *PCCB* (OMIM #232000) genes encoding the enzyme propionyl-CoA carboxylase. MMA is caused by pathogenic variants of the *MUT* gene (OMIM #609058) encoding methylmalonyl-CoA mutase. These enzymes participate in the catabolism of branched-chain amino acids, synthesis of propionyl-CoA by normal gut flora, and breakdown of odd chain fatty acids [3]. A defect in their function will result in the accumulation of toxic levels of propionyl-CoA and methylmalonyl-CoA, which can cause metabolic decompensation, a common characteristic of both MMA and PA.

Various mechanisms have been proposed for hyperammonemia in patients with PA and MMA. The accumulation of propionyl-CoA or methylmalonyl-CoA results in the competitive inhibition of N-acetyl glutamate synthase (NAGS) and carbamoylphosphate synthase, causing a disruption in the urea cycle and consequently inducing hyperammonemia [4]. High concentrations of 2-methylcitric acid inhibit the function of glutamine synthase essential for ammonia detoxification, which results in an increase in ammonia levels and a decrease in glutamine concentrations [5]. Elevated levels of ammonia can damage the developing brain, induce astrocyte swelling, increase nitric oxide levels, suppress ATP synthesis, and increase free radicals leading to vasogenic edema and cell death [6].

The management of PA and MMA focuses mainly on decreasing the accumulation of toxic substances, which can be achieved in two ways; first, by restricting dietary protein, thereby preventing protein catabolism and decreasing their production, or by decreasing the production of propionyl-CoA by the gut flora using antibiotics [7]. Second, by increasing the excretion of toxic metabolites through the administration of l-carnitine, which converts propionyl-CoA and methylmalonyl-CoA to non-toxic propionylcarnitine and methylmalonylcarnitine, respectively [8]. l-Carnitine also replenishes intracellular carnitine stores [9]. An alternative approach is to increase enzyme levels through liver transplantation. During acute decompensation, hyperammonemia can be managed using carglumic acid (N-carbamylglutamate), a structural analog of NAG, which stimulates carbamoyl phosphate synthase and promotes the removal of ammonia via the urea cycle [10, 11].

The effects of carglumic acid on reducing ammonia levels and decompensation episodes in PA and MMA have been well established [12]. However, this approach usually starts after the onset of hyperammonemia, with long-term detrimental consequences on the nervous system. There is currently no quality evidence supporting the long-term use of carglumic acid in PA and MMA.

Here, we report the results of what we believe to be the first clinical trial to evaluate the efficacy of long-term administration of carglumic acid, in addition to standard treatment for the management of PA and MMA.

## Methods

The clinical trial protocol has been previously published [6]. There were no changes or deviations from the protocol when the trial was underway. The trial was investigator-initiated, and the principal investigator was responsible for the design and conduct of the study. This research was funded by Recordati Rare Diseases, which provided trial medication.

The study was conducted per the latest version of the Declaration of Helsinki and the International Conference on Harmonization (ICH) on Good Clinical Practice (GCP) guidelines. This study was approved by the institutional review boards (IRB) of the participating centers and by the Saudi Food and Drug Authority (SFDA) (33066). Under the short title of “Carglumic Acid in Methylmalonic Acidemia and Propionic Acidemia (CAMP),” the study was registered in ClinicalTrial.gov, identifier: NCT02426775.

The study design was a prospective, multicenter, randomized, parallel-group, open-label, controlled clinical trial. This study aimed to compare the efficacy of adding carglumic acid (50 mg/kg/day in divided doses, twice daily) to standard treatment in patients with PA and MMA. The study was conducted between November 12, 2015, and March 27, 2019, in two tertiary care centers in Riyadh, Saudi Arabia, to recruit a minimum of 28 patients, with 14 patients for each disease. Patients were randomized to receive either carglumic acid in addition to standard treatment or standard treatment alone. Although a placebo-controlled study would have been ideal, emergency management of acute crises using carglumic acid as a rescue medication would have been difficult because of blinding.

Each patient was followed up for 24 months, during which they were assessed in six follow-up visits in addition to one visit after the completion of the study for those receiving carglumic acid.

The study included patients aged ≤ 15 years whose parents or legal guardians had provided written consent and were not participating in any other trial. PA was confirmed by measuring acylcarnitine profile, urine organic acid, propionyl-CoA carboxylase in leukocytes or cultured fibroblasts, or by DNA molecular testing of the *PCCA* or *PCCB* genes. MMA was confirmed by measuring acylcarnitine profile, urine organic acid, methylmalonyl-CoA mutase in cultured fibroblasts, or DNA molecular testing of the *MUT* gene. We only included patients with an expected survival of ≥ 6 months. These were defined as those not admitted to the pediatric intensive care unit (PICU) for > 2 times/year because of hyperammonemia, asymptomatic patients diagnosed by newborn screening, or stable chronic patients who were followed up at the outpatient clinic. Genotyping was performed on all participants to confirm the diagnosis. Exclusion criteria included patients with other organic acidemias or hyperammonemia owing to other causes, those receiving other investigational therapy for PA or MMA, or had PA or MMA in addition to other inherited anomalies.

The study included two arms: a standard (control) arm, in which the patients received only the standard treatment, and a carglumic acid arm, in which the patients received carglumic acid in addition to standard treatment. Standard treatment followed the most recent guidelines [6, 13, 14] of l-carnitine (150 mg/kg/day divided and given every 8 h), metronidazole (15 mg/kg/day divided and given every 8 h for one week each month), and a protein-restricted diet. Carglumic acid was administered at 50 mg/kg/day, twice daily in divided doses with meals. All treatments were administered to patients enterally, orally, by a nasogastric tube, or by a gastrostomy tube. At each follow-up visit, patients underwent a complete clinical evaluation, a tally of the previous emergency room admissions because of hyperammonemia, a review of their medication, and appropriate laboratory tests. Hyperammonemia was defined according to age [15]. The dietary management aimed to ensure proper weight gain, linear growth, and head growth. Normal psychomotor development was assessed using serial examinations and valid developmental screening tools.

Natural proteins were restricted in the diet to provide limited amounts of isoleucine, methionine, threonine, valine, and odd chain fatty acids, and the diet was supplemented with metabolic formulas (PROPIMEX®-1 or 2 according to age) to provide additional amino acids and avoid deficiencies of essential amino acids, fatty acids, and micronutrients. The metabolic dietitian ensured the home formula supplies included metabolic formula PROPIMEX®-1 or 2 (according to age) [16, 17]. For infants, breast milk or a regular formula is a natural protein source. For older children, a balanced diet is the natural source of protein.

The mothers with weighing scales, appropriate preparation of formula, and 3-day diet records before clinic visit varied from patient to patient, which affected the monitoring of amino acid concentrations, prealbumin, albumin, and other biochemical parameters according to the protocol [18–20]. The nutritional management plan was the same for all patients at all participating centers. Fasting was prohibited for patients to maintain adequate hydration. Finally, aggressive medical and nutrition interventions (sick day formula) were performed during critical illnesses. On the basis of clinical and biochemical monitoring, the amount of natural protein required must be calculated on a case-by-case basis.

Plasma glycine, isoleucine, methionine, threonine, and valine concentrations were monitored regularly according to the manufacturer’s protocol. In case of low protein levels, the prescribed amount of natural protein was increased by 10–25%, and the patient was re-evaluated by measuring the plasma concentration in 3 days. This process was repeated until the value was in the treatment range. The growth, weight, and height were monitored at every visit to the nutritionist’s clinic. If the patient remained below the usual growth range and did not respond to an increase in protein and energy, or could not consume the prescribed diet via oral, nasogastric, or gastrostomy tube feeding.

The primary outcome was to determine the long-term effectiveness (2 years) of 50 mg/kg/day of carglumic acid, twice daily, in addition to standard treatment in reducing the number of ER admissions because of hyperammonemia in patients with PA or MMA compared with that of standard therapy alone. Secondary outcomes included comparing effects on plasma ammonia levels from baseline to the end of the study, time to the first episode of hyperammonemia, levels of relevant biochemical biomarkers including plasma amino acids, acylcarnitine profile, and urine organic acids, and the number of hospitalization days. The study was monitored by an independent safety monitoring board, which conducted an interim analysis and reviewed the safety data.

### Sample size calculation

#### Base model (with no covariates)

From the previous historical data from the participating centers, the expected baseline rate of events for standard treatment is approximately six events per year. For a total sample size of 18 subjects (9 control and 9 diseased), we assumed the study to have a one-sided hypothesis with a power of 80% to detect a 30% reduction in events over the 2-year study duration with a type I error of 5%. All calculations were performed using the Signorini software [21].

### Model with covariates

To determine the maximum sample size needed for the study in the presence of covariates, the sample size was estimated using the variance inflation factor technique [22, 23]. It inflates the sample size in proportion to the correlation between the covariates and the main effect. The power analysis focused on whether the condition was PA or MMA, as this is one of the main covariates that could be imbalanced between treatment arms. Assuming that the condition type variable can explain 24% of the observed variation in the study arms, we estimated the effective sample size needed for the study to be 24 subjects. Assuming a 10% dropout rate, the expected total sample size needed for the study was 28 subjects.

### Randomization

The randomization process used an automated web-based system with a variable block size selected to ensure that the study groups were balanced given the small number of patients. Subjects were randomized after accounting for the stratification variables—the condition (PA or MMA) and the number of ER admissions before randomization (0 admissions, 1–5, and > 5 admissions).

### Blinding

The study was open-label as patients might present to the ER with acute crises, where the attending physicians would need the details of their treatment regimens. Additionally, according to the emergency protocol for the management of PA and MMA, carglumic acid is considered a rescue medication and should be used in all patients with hyperammonemia, even for those in the standard treatment arm.

### Statistical analysis

The primary endpoint was compared across the study arm using Poisson regression. For subjects with less than 24 months of follow-up because of dropout, an offset variable of the actual follow-up duration was included in the model. The model was assessed for over-dispersion, and a robust variance estimator was used to minimize the impact of model misspecification on the type I error [24]. Results were reported in terms of the rate ratios with the corresponding 95% confidence intervals. All results were declared significant, with a type I error of 0.05. Secondary endpoints, such as the time-to-first event between the two groups, were summarized and reported using a Kaplan–Meier curve. The rates of the events were compared using the log-rank test.

## Results

**Fig. 1:**
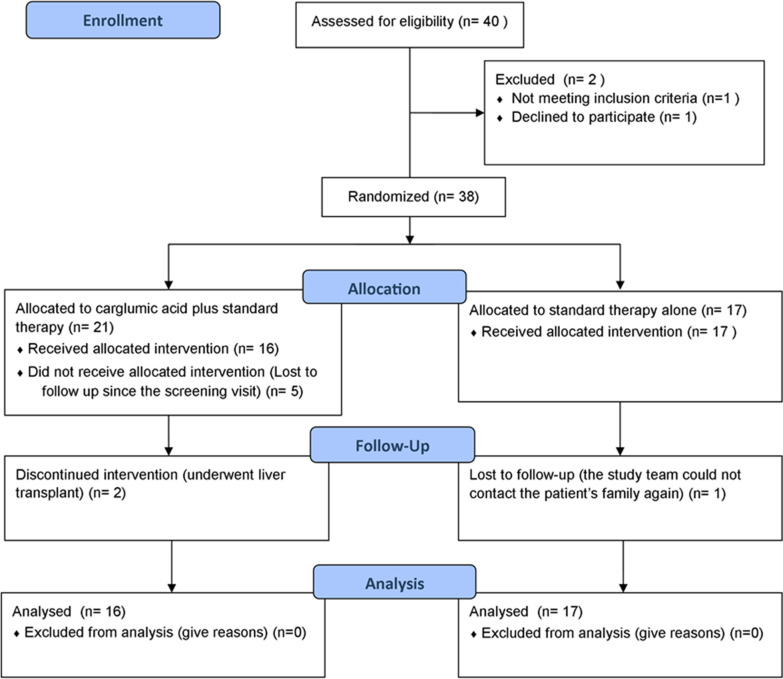
CONSORT flow diagram: Patient recruitment and flow during the trial

Trial recruitment started in November 2015 and continued until April 2019, when the required number of participants was reached. Forty patients were screened during this period (Fig. 1). Twenty-one patients were randomized to the carglumic acid arm, and 17 were allocated to the standard therapy arm. Of the 21 patients allocated to receive carglumic acid, 5 did not return after the screening visit and were excluded from the analysis. During the trial, two patients in the carglumic acid arm underwent liver transplantation and discontinued the follow-up visits; however, their data were included in the final analysis. Although one patient from the standard treatment arm was lost to follow-up after four visits, their data were included in the final analysis.

The randomized patients included 27 boys and 11 girls, almost equally distributed between the two arms (p = 0.721). The mean age of the participants in the standard treatment arm was approximately 36 months, while the mean age in the carglumic acid arm was approximately 40 months. All other demographic characteristics between the two arms were evenly distributed (Additional File 1: Table S1). The baseline Z-scores of the growth parameters and the growth velocity of the patients throughout the study are presented in Additional file 1: Tables S2 and S3, respectively. confirmed mutations in the patients, and the previous number of ER admissions are presented in Additional file 1: Table S4 and an excel sheet contain all nutritional data and showed that there was no significant change in total Protein per kg/day between the two treatment groups (p = 0.062). In the interaction effect (p = 0.6158), there was no significant change between the two treatment groups in total Protein per kg/day over time.

Compliance with the study drug was assessed by the study coordinator at each visit. The data were recorded in the CRF for any missed doses, the reason for missing doses, any adverse effects, and any associated issues beginning at the first visit. Additionally, a pill count was performed at each visit, and the number of remaining tablets was compared with the theoretical count. The patients were asked to return the empty or expired medication to the dispensing pharmacy. The compliance with the study medication was satisfactory. Missed doses were very limited in the carglumic acid arm. The main reason for the difference in counts was the admission to the hospital during which patients received the emergency dose of carglumic acid.

### Primary outcome

**Fig. 2:**
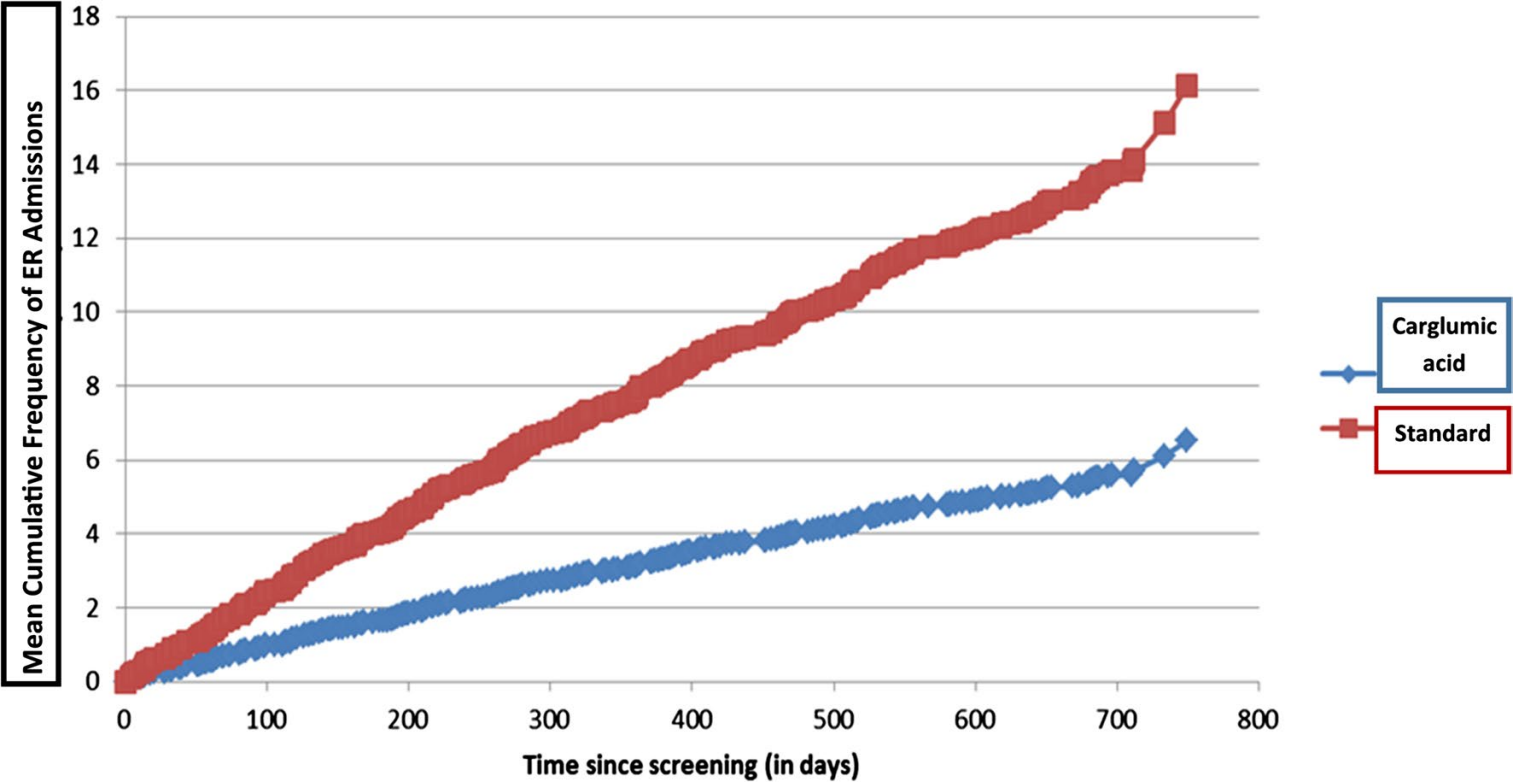
Mean cumulative emergency room admission frequency between the two arms

The total number of ER admissions was 12.76 in the standard treatment group and 6.31 in the carglumic acid group. Results of the Poisson regression analysis suggest that carglumic acid achieved a significant 51% (0.4945, [0.2904, 0.8422]) reduction in the number of ER admissions during the 2-year observation period compared with standard therapy (p = 0.0095) (Table 1). These results were robust and were not significantly altered across major factors such as sex, age, and disease type (Table 2). Figure 2 shows the mean cumulative ER admissions frequency between the two arms throughout the study.

**Table 1:** Primary outcome results

	Overall	Standard therapy N = 17	Carglumic acid N = 16
Mean total ER visits (STD)	9.64 ± 9.12	12.76 ± 11.18	6.31 ± 4.61
Min	0.00	0.00	0.00
Max	41.00	41.00	16.00
Total ER visits			
0 visits n (%)	5 (15.15)%	3 (17.65%)	2 (12.50%)
1–2 visits n (%)	3 (9.09)%	1 (5.88%)	2 (12.50%)
3–4 visits n (%)	4 (12.12)%	2 (11.76%)	2 (12.50%)
5–6 visits n (%)	4 (12.12)%	1 (5.88%)	3 (18.75%)
More than 6 Visits n (%)	17 (51.52)%	10 (58.82%)	7 (43.75%)

**Table 2:** Poisson regression analysis of the rate of ER visits (intention-to-treat population)

	Rate ratio	95% LCL	95% UCL	P-value
Treatment				
*Carglumic acid versus standard therapy	0.4945	0.2904	0.8422	0.0095
Treatment				
**Carglumic acid versus standard therapy	0.4991	0.3017	0.8256	0.0068
Gender				
Male versus female	0.5834	0.2864	1.1884	0.1377
Disease type				
MMA versus PA	0.8340	0.4416	1.5752	0.576
Age in years	0.9959	0.9845	1.0073	0.4795

*Poisson model including treatment only as an independent variable

**Poisson model including treatment, gender, disease type, and age as an independent variable

### Secondary outcomes

The plasma ammonia levels from the baseline and at the end of the study did not show any significant difference between the arms (Additional file 2: Fig. S2). When the times of the first episode of hyperammonemia were compared using the Kaplan–Meier curve, both arms had a comparable course (Additional file 2: Fig. S3–S5). For the biochemical markers (Table 3), there was a significant difference in the level of plasma glycine favoring the carglumic acid arm (p = 0.046). There were no significant differences in the levels of other plasma amino acids. A significant difference in free carnitine levels between the two arms (p = 0.0376) was observed (Additional file 1^1^ Table S5). There were no significant differences in total carnitine or acylcarnitine levels between the groups. The urine organic acid levels in the two arms were comparable for both the diseases (Additional file 1: Table S5). Although patients in the carglumic acid arm reported numerically fewer days of admission than those in the standard treatment (32.8 and 51.29 days, respectively), the difference was not significant (p = 0.4) (Additional file 1: Table S6).

**Table 3:** Secondary outcome results

Biomarker	Relative change	95% LCL	95% UCL	p-value
Ammonia: Carglumic acid versus standard	0.868924	0.725061	1.041435	0.1283
Glycine: Carglumic acid versus standard	0.748039	0.561861	0.995809	0.0468
Methionine: Carglumic acid versus standard	1.004108	0.861483	1.17023	0.9585
Leucine: Carglumic acid versus standard	0.89682	0.720219	1.116836	0.3306
Isoleucine: Carglumic acid versus standard	0.952276	0.680859	1.331891	0.7753
Valine: Carglumic acid versus standard	0.918972	0.753746	1.120416	0.4035
Threonine Carglumic acid versus standard	1.02429	0.781219	1.342992	0.8623
Glutamine Carglumic acid versus standard	0.965316	0.820534	1.135644	0.6704

### Safety results

No serious adverse events were reported during the study period. The only two reported adverse events were allergy in one patient, who was pre-randomized to the standard arm and continued in the study, and mild vomiting in another after the administration of the study medication. It was resolved by advising the mother to administer the dose slowly.

All vital signs, physical examination, and routine laboratory data were comparable between the study arms (Additional file 1: Tables S7–S13).

## Discussion

PA and MMA are considered ultra-rare metabolic diseases with poor prognosis and severe course of the disease, including permanent neurological sequelae in cases of late diagnosis or lack of early intervention. Therefore, newborn screening programs have been implemented worldwide to identify affected individuals at the asymptomatic stage in early infancy [6]. The treatment of these diseases is multidisciplinary; however, most guidelines focus on managing acute hyperammonemia. The management depends on the ammonia levels during the decompensation episodes and the response to first-line medications, ranging from using nitrogen scavengers, carglumic acid, and carnitine to continuous hemodiafiltration. Long-term management focuses mainly on a protein-restricted diet while keeping an eye on the patients’ daily needs for normal growth and development and administration of l-carnitine and metronidazole [25].

Liver transplantation is another alternative for long-term management to prevent decompensations and improve the quality of life [6]. However, a critical evaluation of the patient’s condition, including the response to medical treatment and the current developmental and neurological status, is needed to weigh the benefits versus the risks of a transplant [26]. Moreover, as PA and MMA are multiorgan diseases, medical treatment is the first-line choice for their management.

To the best of our knowledge, the current clinical trial is the first randomized controlled trial to study the long-term effectiveness of adding carglumic acid to the treatment regimen for PA and MMA patients. A randomized controlled trial is a gold standard trial for establishing effectiveness in a research setting. The study design was chosen to be a parallel-group instead of a crossover design. Although the rarity of the diseases under study and the variability of the phenotype could make the crossover design a superior option, to maintain the statistical power of the study, the duration of the trial had to be prolonged up to 8 years. Such a long study was not feasible at the participating centers, and there was a higher possibility of participants dropping out. To alleviate the effect of heterogeneity of the participants resulting from the study design, the previous number of ER admissions (Additional file 1: Table S1 and S4 and excel sheet contain all nutritional data) for each patient was taken into consideration during the randomization process to maintain a balance between the two arms. Variable block randomization was selected to ensure that the study groups were balanced, given the small number of patients. However, this approach increases the complexity of statistical analysis. Moreover, this design could result in a serious imbalance in the study groups in case of frequent withdrawal of the patients during the study. Therefore, we planned to perform the analysis twice, first with the data for all the patients, including those withdrawn or lost to follow-up (intention to treat) in the statistical analysis, and then only with the data from the participants who completed the study. The results of both analyses were comparable, indicating that the withdrawals did not affect the balance in the study arms.

In the USA, carglumic acid is approved for the treatment of acute and chronic hyperammonemia due to NAGS deficiency. In the EU, it is approved for the treatment of hyperammonemia due to NAGS deficiency, Isovaleric acidemia (IVA), PA, or MMA, including long-term treatment. Carglumic acid has been successful in the management of acute hyperammonemia in PA and MMA [10, 27–32]. It increases ureagenesis, a targeted treatment for hyperammonemia under various conditions [33]. In patients with PA, carglumic acid stimulated ureagenesis and decreased plasma ammonia levels [34]. The European Medicines Agency (EMA) approved the use of carglumic acid in organic acidemias based on a multicenter retrospective study of 41 patients with decompensation episodes secondary to PA (39%), MMA (51.2%), and IVA (9.8%) treated with carglumic acid with and without ammonia-scavenging drugs for an average of 5.5 days. In 2016, an Italian group reported their clinical experience of using a 50 mg/kg/day dose of carglumic acid in the management of PA and MMA. Their results showed that in addition to being efficacious for the treatment of acute hyperammonemia, carglumic acid was also effective and well-tolerated as a long-term treatment in patients with severe PA and MMA [35]. Our results support these previous studies in proving the benefit of carglumic acid in decreasing the frequency of hyperammonemic decompensations over the long term.

In the secondary analysis, the levels of ammonia were comparable between the two study arms. However, this could be explained by the timing of sample collection, which was preplanned to be taken during the routine follow-up visits in the outpatient setting and not during the decompensations. Although free carnitine was significantly lower in the carglumic acid arm, both readings were within the normal range. Additionally, all patients were on carnitine supplementation, which might complicate the explanation of this result. Nevertheless, one explanation is that in the carglumic acid arm, patients were using more carnitine in the anabolic process because of the improvement in their metabolism.

Hyperglycinemia in PA and MMA could be attributed to the inhibition of glycine cleavage by propionyl-CoA [36]. The significant reduction in the level of glycine in the carglumic acid arm suggests an improved elimination of toxic metabolites. Further studies are needed to explain the mechanism of glycine reduction in patients with carglumic acid.

The number of days of hospitalization was lower in the carglumic acid arm than in the standard arm; however, this difference was not statistically significant. The duration of admission to the hospital might not be an ideal element to evaluate the metabolic status of the patients, unlike episodes of hyperammonemia and ER admissions, because a wide variety of causes unrelated to the primary disease may extend the duration of admission. We noticed variable causes for prolonged admission to the patients in general, including but not limited to routine radiological investigations, interventions related to the gastrostomy tubes and central lines, social reasons as some of the patients were living far away from the hospital, and nosocomial infections. These causes could not be evaluated further in the current study and may require further studies for critical evaluation.

The most common adverse events previously reported for carglumic acid include infections, vomiting, abdominal pain, pyrexia, tonsillitis, anemia, ear infection, diarrhea, nasopharyngitis, and headache [36]. During the study, the medication was well-tolerated by the patients. The only reported adverse event was a mild allergic reaction in the form of perioral erythema and vomiting, which was also mild and managed with slower administration.

This study has certain limitations. and gaps and caution should be used when interpreting the results. The heterogeneity of the patients may have confounded the results. Additionally, the reduced number of ER visits because of hyperammonemia may not necessarily correlate with significant clinical outcomes. Therefore, the study has a high risk of information and selection biases that may threaten internal and external validity. The main limitation was the limited number of participants, mainly because of the ultra-rare nature of the diseases under study. The phenotypic variation between the two arms was another challenge; however, the unique randomization plan alleviated the effect of this variation. Being an open-label trial was another limitation; however, the patients' safety and well-being were prioritized as blinding may affect the quality of health care management for the patients without adding a significant benefit to the design or the outcome of the study. Additionally, this trial was initiated, for which getting a placebo and, accordingly, being able to blind the patients and the investigators was not feasible. The heterogeneity of the patient population could have a negative impact on the trial design in two ways; first, if the groups are imbalanced across the study arms, they could influence the results, and second, the inclusion of the two conditions could cause increased variability and could lead to an underpowered study. To ensure that the two groups are balanced between the two study arms, we randomized the patients by blocking on the PA and MMA. The baseline clinical characteristics indicated that the two groups were balanced between the study arms. To further examine the effect of disease subgroups, we included indicator variables in the regression model. The results suggested that the disease subtype had a minimal impact on the outcome. We also assessed the variability introduced by combining those conditions on the power analysis during the design phase of the trial, and the study was powered accordingly.

A higher-than-necessary protein intake can lead to an increase in the frequency of decompensations. This trial may have a larger number of admissions as a result of this fact, but this could be read to mean that carglumic acid has the ability to boost protein tolerance. More research is required to investigate this theory.

In conclusion, this is the first randomized controlled clinical trial to evaluate the effectiveness of long-term use of carglumic acid in patients with PA and MMA. Carglumic acid was found to be safe and well-tolerated over the long term. There was a significant decrease in the number of ER admissions because of hyperammonemia in the carglumic acid arm compared to that in the standard arm. This decrease in the number of ER admissions could improve the quality of life of patients and their families.

### Web resources

ClinicalTrials.gov, https://clinicaltrials.gov

European Medical Agency, https://www.ema.europa.eu/en/documents/variation-report/carbaglu-h-c-461-ii-0013-epar-assessment-report-variation_en.pdf

FDA, https://www.accessdata.fda.gov/drugsatfda_docs/nda/2010/022562s000TOC.cfm

FDA, https://www.accessdata.fda.gov/drugsatfda_docs/label/2010/022562lbl.pdf

OMIM, https://www.omim.org/.

## Supplementary Information

**Additional file 1. Table S1**: Demographic data, growth parameters and patient characteristics. **Table S2**: The baseline growth Z-scores between the two groups. **Table S3**: The growth velocity throughout the trial between the two study groups. **Table S4 and an excel sheet**: showed Nutritional management, Molecular results, and previous ER admissions. **Table S5**: Acylcarnitine profile and urine organic acids results. **Table S6**: Number of days of hospitalization. **Table S7**: Lab results at baseline (Visit 1). **Table S8**: Lab results for Visit 2 (3 months ± 14 days). **Table S9**: Lab results for Visit 3 (6 months ± 14 days). **Table S10**: Lab results for Visit 4 (9 months ± 14 days). **Table S11**: Lab results for Visit 5 (12 months ± l4 days). **Table S12**: Lab results for Visit 6 (18 months ± 14 days). **Table S13**: Vital signs as well as the growth parameters.

**Additional file 2. Fig. S1**: The Emergency room admissions distribution among the two diseases in the two arms of the study. **Fig. S2** The Emergency room admissions distribution between the two arms of the study. **Fig. S3** Distribution of ammonia levels over time. The arms showed comparable ammonia levels throughout the study. **Fig. S4** Ammonia level distribution between the two groups throughout the study visits. **Fig. S5** Kaplan–Meier plot to evaluate the time to first emergency room visit between the study groups. The plot did not show any statistically significant difference.

**Additional file 3. Fig. S6**: The average total protein intake per KG per day for the two groups over time.

## Data Availability

The datasets during and/or analyzed during the current study available from the corresponding author on reasonable request (dralfadhelm@gmail.com).

## Abbreviations

PA: Propionic acidemia (PA)
MMA: Methylmalonic academia
ER: Emergency room
NAGS: *N*-Acetyl glutamate synthase
PICU: Pediatric intensive care unit
EMA: European medicines agency
KFMC: King Fahad Medical City
KAMC: King Abdulaziz Medical City
