# Supplementary material for: Long-term effectiveness of carglumic acid in patients with propionic acidemia (PA) and methylmalonic acidemia (MMA): a randomized clinical trial
Source: Orphanet J Rare Dis. 2021 Oct 11;16:422. doi: 10.1186/s13023-021-02032-8 (PMC8507242; doi:10.1186/s13023-021-02032-8)
Supplement: Supplementary file 1 — Additional file 1. Table S1: Demographic data, growth parameters and patient characteristics. Table S2: The baseline growth Z-scores between the two groups. Table S3: The growth velocity throughout the trial between the two study groups. Table S4 and an excel sheet: showed Nutritional management, Molecular results, and previous ER admissions. Table S5: Acylcarnitine profile and urine organic acids results. Table S6: Number of days of hospitalization. Table S7: Lab results at baseline (Visit 1). Table S8: Lab results for Visit 2 (3 months ± 14 days). Table S9: Lab results for Visit 3 (6 months ± 14 days). Table S10: Lab results for Visit 4 (9 months ± 14 days). Table S11: Lab results for Visit 5 (12 months ± l4 days). Table S12: Lab results for Visit 6 (18 months ± 14 days). Table S13: Vital signs as well as the growth parameters. [file 13023_2021_2032_MOESM1_ESM.docx]

**Supplementary Material**

- Table S1: Demographic data, growth parameters and patient characteristics:
- Table S2: The baseline growth Z-scores between the two groups.
- Table S3: The growth velocity throughout the trial between the two study groups.
- Table S4: Molecular results, and previous ER admissions
- Table S5: Acylcarnitine profile and urine organic acids results
- Table S6: Number of days of hospitalization
- Table S7: Lab results at baseline (Visit 1)
- Table S8: Lab results for Visit 2 (3 months±14 days)
- Table S9: Lab results for Visit 3 (6 months±14 days)
- Table S10: Lab results for Visit 4 (9 months±14 days)
- Table S11: Lab results for Visit 5 (12 months±l4 days)
- Table S12: Lab results for Visit 6 (18 months±14 days)
- Table S13: vital signs as well as the growth parameters
- Figure S1: The Emergency room admissions distribution among the two diseases in the two arms of the study.
- Figure S2: The Emergency room admissions distribution between the two arms of the study.
- Figure S3: Distribution of ammonia levels over time. The arms showed comparable ammonia levels throughout the study.
- Figure S4: Ammonia level distribution between the two groups throughout the study visits.
- Figure S5: Kaplan-Meier plot to evaluate the time to first emergency room visit between the study groups. The plot did not show any statistically significant difference.
- Additional file 3: **Fig.S6** the average total protein intake per KG per day for the two groups over time.
- Excel sheet contain all nutritional data.

Table S1: Demographic data, growth parameters and patient characteristics:

|  | | Carglumic acid  N=21 | Standard therapy  N=l7 | P-value |
| --- | --- | --- | --- | --- |
| Age in months Mean±STD | | 40.37±32.53 | 35.83±36.59 |  |
| Min | | 1.17 | 0.33 | 0.5279 |
| Max | | 110.37 | 121.89 |  |
| Gender | |  |  |  |
| Male n (%) | | 14 (66.67%) | 13 (76.47%) | 0.7210 |
| Female n (%) | | 7 (33.33%) | 4 (23.53%) |  |
| Family History  Yes n (%) | | 9 (42.86%) | 8 (47.06%) | 1.0000 |
|  | Metronidazole |  |  |  |
|  | No n (%) | 9 (42.86%) | 9 (52.94) | 0.7446 |
| Standard | Yes n (%) | 12 (57.14%) | 8 (47.06) |  |
| treatment |  |  |  |  |
|  | Carnitine |  |  |  |
|  | No n (%) | 0 (0.00%) | 1 (5.88%) | 0.4474 |
|  | Yes n (%) | 21 (100.00%) | 16 (94.12%) |  |
| Mean systolic blood pressure ± STD | | 90.85 ± 23.99 | 96 ± 12.08 | 0.9746 |
| Mean diastolic blood pressure ± STD | | 58.05 ± 16.91 | 62.13 ± 10.46 | 0.4071 |
| Heart rate Mean ± STD | | 109.57 ± 28.55 | 117.56 ± 11.22 | 0.3985 |
| Respiratory rate ± STD | | 24.33 ± 11.37 | 25.81 ± 5.02 | 0.1201 |
| Number of prior hospitalizations categories | Zero | 2 (9.52%) | 1 (5.88%) | 1.0000 |
|  | 1 to 5 | 13 (61.90%) | 11 (64.71%) |  |
|  | more than 5 | 6 (28.57%) | 5 (29.41%) |  |
| Number of Previous ER admissions (6 months prior to randomization)    Total  Mean ± STD  Median | | 62  3.6471 ± 2.2344  4 | 67  4.1875± 3.1245  3 | 0.5699 |
| Disease Type n (%) | |  |  |  |
| PA | | 12 (57.14%) | 9 (52.94%) | 1.0000 |
| MMA | | 9 (42.86%) | 8 (47.06%) |  |

Table S2: The baseline growth Z-scores between the two groups.

| Variable | Carglumic acid | Standard | P value |
| --- | --- | --- | --- |
| Weight | -2.41±2.04 | -0.52±1.49 | 0.0085 |
| Hight | -1.51±1.24 | -0.32±2 | 0.0610 |
| Head  circumference | -0.59±0.8 | 0.03±2.09 | 0.4009 |

Table S3: The growth velocity throughout the trial between the two study groups.

| Variable | Carglumic acid | Standard | P value |
| --- | --- | --- | --- |
| Height (cm) | 12.12±8.02 | 13.46±7.46 | 0.6208* |
| Weight (kg) | 3.66±2.01 | 3.67±1.82 | 0.9904* |
| Head  Circumference  (cm) | 3.52±3.39 | 2.29±2.24 | 0.3103** |

Reported statistics are Mean ± Standard Deviation

**T test*

***Wilcoxon tow sample test*

| Variable | Carglumic acid | Standard | P value |
| --- | --- | --- | --- |
| Height (cm) | 10(10.75) | 13(8) | 0.6208* |
| Weight (kg) | 3.45(2.55) | 3.8(3.3) | 0.9904* |
| Head  Circumference  (cm) | 1.85(5) | 2(3.3) | 0.3103** |

Reported statistics are Median (IQR)

**T test*

***Wilcoxon tow sample tes*

Table S4: Molecular results, and previous ER admissions

| **Randomization ID*** | **Gene** | **Mutation** | **Number of previous ER Admissions**** |
| --- | --- | --- | --- |
| 1 | PCCA | c.425G>A (p.Gly142Asp) | 2 |
| 2 | MUT | c.329A>G (p.Tyr110Cys) | 2 |
| 3 | PCCB | c.1050dupG (p.Thr351AspfsTer10) | 10 |
| 4 | PCCA | c.350G>A (p.Cys117Tyr) | 1 |
| 5 | PCCA | c.425G>A (p.Gly142Asp) | 5 |
| 7 | MUT | c.2200C>T (p.Gln734Ter) | 8 |
| 8 | PCCA | c.425G>A (p.Gly142Asp) | 7 |
| 12 | MUT | c.329A>G (p.Tyr110Cys) | 6 |
| 14 | MUT | c.2200C>T (p.Gln734Ter) | 4 |
| 15 | MUT | c.329A>G (p.Tyr110Cys) | 2 |
| 16 | PCCA | c.425G>A (p.Gly142Asp) | 3 |
| 17 | MUT | c.329A>G (p.Tyr110Cys) | 3 |
| 18 | PCCA | c.425G>A (p.Gly142Asp) | 7 |
| 19 | PCCB | c.990dupT (P.Glu331XfsX1) | 6 |
| 20 | MUT | c.329A>G (p.Tyr110Cys) | 10 |
| 21 | MUT | c.329A>G (p.Tyr110Cys) | 2 |
| 22 | MUT | c.329A>G (p.Tyr110Cys) | 1 |
| 23 | MUT | c.329A>G (p.Tyr110Cys) | 4 |
| 25 | MUT | c.810_811delGGinsA (P.Ala271LeufsX11) | 6 |
| 26 | PCCA | c.425G>A (p.Gly142Asp) | 0 |
| 28 | MUT | c.2200C>T p.Gln734Ter | 1 |
| 30 | PCCA | c.425G>A (p.Gly142Asp) | 3 |
| 31 | PCCA | c.425G>A (p.Gly142Asp) | 5 |
| 32 | PCCA | c.425G>A (p.Gly142Asp) | 2 |
| 34 | PCCA | c.425G>A (p.Gly142Asp) | 3 |
| 35 | PCCA | c.425G>A (p.Gly142Asp) | 0 |
| 36 | MUT | c.2200C>T (p.Gln734Ter) | 5 |
| 37 | PCCA | c.425G>A (p.Gly142Asp) | 4 |
| 38 | PCCA | c.1288C>T (p.Arg430Ter) | 6 |
| 40 | MUT | c.1052G>T (p.Cys351Phe) | 5 |
| 41 | MUT | c278G>A (p.Arg93His) | 2 |

* The data of the withdrawn patients was not presented in this table.

** The ER admissions for each patient for 6 months prior to randomization.

Table S5: Acylcarnitine profile and urine organic acids results:

| Variable | Treatment Arm | | | p-value |
| --- | --- | --- | --- | --- |
|  | Carglumic acid | Standard treatment | |  |
| Total camitine (Mmol/L) | 86.18 ± 25.25 | 106.72 ± 44.8 | | 0.1692 |
| Free carnitine (Mmol/L) | 37.38± 28.62 | 39.02 ± 11.92 | | 0.0376 |
| Urine methylmalonic acid (umol/mmol  create), MMA | 1911 ± 1437.39 | 4856.97 | ± 3415.61 | 0.2361 |
| Urine hydroxypropionic acid  (umol/mmol create), PA | 308.48 ± 225.74 | 275.55 ± 487.56 | | 0.2774 |

Reported statistics are mean ± standard deviation, Wilcoxon two-sample test for association.

Table S6: Number of days of hospitalization

|  | Carglumic acid  N=16 | Standard therapy  N=17 | P-value |
| --- | --- | --- | --- |
| Total number of days of hospitalization |  |  |  |
| Mean ± STD | 32.8±30.60 | 51.29±44.87 |  |
|  |  |  | 0.4061 |
| Min | 0.00 | 0.00 |  |
|  |  |  |  |
| Max | 116.00 | 118.00 |  |

Table S7: Lab results at baseline (Visit 1)

|  | Carglumic acid  N=21 | Standard therapy  N=17 | | P-value | |
| --- | --- | --- | --- | --- | --- |
| PT (Sec)  Up normal n(%) | 8 (47.06%) | 6 (54.55%) | | 1.0000 | |
| APTT (sec)  Up normal n(%) | 8 (47.06%) | 7 (63.64%) | | 0.4601 | |
| INR  Up normal n(%) | 10 (58.82%) | 9 (81.82%) | | 0.2495 | |
| Glucose level (mmol/L) Mean ± STD | 4.49±0.9 | 5.09±1.07 | |  | |
| Min | 3.10 | 3.10 | | 0.0935 | |
| Max | 6.00 | 7.80 | |  | |
| Urea level (mg/dL) Mean ± STD | 4.98±2.54 | 5.68±2.91 | |  | |
| Min | 1.20 | 1.40 | | 0.3233 | |
| Max | 11.00 | 14.00 | |  | |
| Creatinine level (Mmol/L) Mean ± STD | 35.95±l5 | 38.67±15.43 | |  | |
| Min | 14.00 | 15.00 | | 0.6039 | |
| Max | 73.00 | 81.00 | |  | |
| Uric Acid level (Mmol/L) Mean ± STD | 220.86±111.37 | 211.91±48.62 | |  | |
| Min | 72.00 | 152.00 | | 0.7631 | |
| Max | 441 .00 | 290.00 | |  | |
| LDH (U/L) Level Mean ± STD | 207.36±123.4 | 242.57±157.72 | |  | |
| Min | 1.50 | 1.12 | | 0.7911 | |
| Max | 323.00 | 494.00 | |  | |
| CK (U/L) level Mean ± STD | 89±52.7 | 131.75±54.31 | |  | |
| Min | 28.00 | 59.00 | | 0.0569 | |
| Max | 174.00 | 251.00 | |  | |
| AST (U/L) Mean ± STD  Min  Max | 33.1 ±19.02  9.50  85.00 | 38.38±38.32  7.00  170.00 | 0.9055 | |  |
| ALT (U/L) Mean ± STD | 28.52±19.79 | 33.63±33.56 |  | |  |
| Min | 7.30 | 7.00 | 0.9076 | |  |
| Max | 94.00 | 156.00 |  | |  |
| LDL (mmol/L) Mean ± STD | 1.57±0.92 | 1.56±0.59 |  | |  |
| Min | 0.53 | 1.01 | 0.7467 | |  |
| Max | 4.17 | 3.48 |  | |  |
| HDL (mmol/L) Mean ± STD | 1.22±0.24 | 1.14±0.37 |  | |  |
| Min | 0.89 | 0.67 | 0.5281 | |  |
| Max | 1.77 | 1.84 |  | |  |
| Triglycerides (mmol/L) Mean ± STD | 1.03±0.34 | 1.42±0.91 |  | |  |
| Min | 0.46 | 0.59 | 0.2619 | |  |
| Max | 1.63 | 4.50 |  | |  |

Table S8: Lab results for Visit 2 (3 months ±14 days)

|  | Carglumic acid  N=16 | Standard therapy  N=17 | P-value |
| --- | --- | --- | --- |
| PT (Sec)  Up normal n(%) | 4(36.36%) | 6(54.55%) | 0.6699 |
| APTT (sec)  Up normal n(%) | 4(36.36%) | 9(81.82%) | 0.0805 |
| INR  Up normal n(%) | 9(81.82%) | 9(81.82%) | 1.0000 |
| Glucose level (mmol/L) Mean±STD | 5.67±1.17 | 4.99±1.02 |  |
| Min | 3.70 | 3.30 | 0.1866 |
| Max | 7.20 | 6.70 |  |
| Urea level (mg/dL) Meant±STD | 4.85±3.64 | 8.19±4.74 |  |
| Min | 1.50 | 3.60 | 0.0586 |
| Max | 15.30 | 18.00 |  |
| Creatinine level (Mmol/L) Mean±STD | 35.17±19 | 32.93±9.4 |  |
| Min | 11.00 | 16.00 | 0.7156 |
| Max | 73.00 | 46.00 |  |
| Uric Acid level (Mmol/L) Mean±STD | 236.64±128.16 | 292.42±152.33 |  |
| Min | 60.00 | 132.00 | 0.3558 |
| Max | 454.00 | 689.00 |  |
| LDH (U/L) Level Mean±STD | 216.39±187.25 | 280.5±81.38 |  |
| Min | 1.17 | 133.00 | 0.4796 |
| Max | 342.00 | 378.00 |  |
| CK (U/L) level Mean±STD | 85±41.84 | 120.09±70.76 |  |
| Min | 19.00 | 30.00 | 0.3242 |
| Max | 131.00 | 259.00 |  |
| AST (U/L) Mean±STD | 29.69±18.1 | 32.2±13.89 |  |
| Min | 7.00 | 7.00 | 0.85 l9 |
| Max | 62.00 | 57.00 |  |
| ALT (U/L) Mean±STD | 25.57±8.72 | 23.09±11.66 |  |
| Min | 9.00 | 6.00 | 0.4761 |
| Max | 42.00 | 43.00 |  |
| LDL (mmol/L) Mean±STD | 1.58±0.66 | 1.47±0.43 |  |
| Min | 0.68 | 1.08 | 0.6935 |
| Max | 3.08 | 2.52 |  |
| HDL (mmol/L) Mean±STD | 1.03±0.28 | 1.08±0.24 |  |
| Min | 0.52 | 0.70 | 0.5112 |
| Max | 1.62 | 1.40 |  |
| Triglycerides (mmol/L) Mean±STD | 0.78±0.29 | 1.51±1.01 |  |
| Min | 0.29 | 0.60 | 0.0412 |
| Max | 1.16 | 3.75 |  |

Table S9: Lab results for Visit 3 (6 months ± 14 days)

|  | Carglumic acid  N=16 | Standard therapy  N=17 | P-value |
| --- | --- | --- | --- |
| PT (Sec)  Up normal n(%) | 7(63.64%) | 6(46.15%) | 0.4442 |
| APTT (sec)  Up normal n(%) | 4(36.36%) | 9(69.23%) | 0.2173 |
| INR  Up normal n(%) | 8(72.73%) | 11(84.62%) | 0.6299 |
| Glucose level (mmol/L) Mean±STD | 5.68±1.94 | 4.49±0.74 |  |
| Min | 3.10 | 3.20 | 0.0449 |
| Max | 9.70 | 6.10 |  |
| Urea level (mg/dL) Mean±STD | 5.96±3.51 | 6.17±4.38 |  |
| Min | 1.20 | 1.60 | 0.708 l |
| Max | 16.20 | 16.30 |  |
| Creatinine level (Mmol/L)  Mean±STD | 39.43±24.32 | 36.06±11.17 |  |
| Min | 13.00 | 16.00 | 0.6621 |
| Max | 104.00 | 54.00 |  |
| Uric Acid level (Mmol/L)  Mean±STD | 277.42±140.96 | 259.31±94.25 |  |
| Min | 58.00 | 67.00 | 0.7071 |
| Max | 549.00 | 463.00 |  |
| LDH (U/L)  Level Mean±STD | 300.29±38.75 | 260.17±21.28 |  |
| Min | 260.00 | 232.00 | 0.0457 |
| Max | 361.00 | 290.00 |  |
| CK (U/L) level Mean±STD | 97±48.34 | 90.18±46.83 |  |
| Min | 40.00 | 9.00 | 0.7464 |
| Max | 182.00 | 165.00 |  |
| AST (U/L) Mean±STD | 32.83±4.2 | 42±22.93 |  |
| Min | 27.00 | 24.00 | 0.6875 |
| Max | 41.00 | 103.00 |  |
| ALT (U/L) Mean±STD | 2 l±6.95 | 26.08±20.93 |  |
| Min | 8.00 | 12.00 | 0.9179 |
| Max | 37.00 | 88.00 |  |
| LDL (mmol/L) Mean±STD | 1.55±0.63 | 1.43±0.52 |  |
| Min | 0.68 | 0.32 | 0.6184 |
| Max | 2.74 | 2.24 |  |
| HDL (mmol/L) Mean±STD | 1.08±0.19 | 1.12±0.29 |  |
| Min | 0.77 | 0.67 | 0.6823 |
| Max | 1.42 | 1.62 |  |
| Triglycerides (mmol/L) Mean±STD | 1.16±1.28 | 1.21±0.58 |  |
| Min | 0.40 | 0.41 | 0.1660 |
| Max | 5.17 | 2.32 |  |

Table S10: Lab results for Visit 4 (9 months ± 14 days)

|  | Carglumic acid  N=16 | Standard therapy  N=16 | P-value |
| --- | --- | --- | --- |
| PT (Sec)  Up normal n(%) | 6(37.5%) | 6(42.86%) | **1.0000** |
| APTT (sec)  Up normal n(%) | 9(56.25%) | 6(42.86%) | 0.7152 |
| INR  Up normal n(%) | 13(81.25%) | 11(78.57%) | 1.0000 |
| Glucose level (mmol/L) Mean±STD | 4.8±l.11 | 4.6l±1.93 |  |
| Min | 3.20 | 1.20 | 0.5187 |
| Max | 7.10 | 9.90 |  |
| Urea level (mg/dL) Mean±STD | 5.49±2.76 | 5.2l±3.2 |  |
| Min | 2.60 | 1.80 | 0.5717 |
| Max | 11.30 | 14.10 |  |
| Creatinine level (Mmol/L) Mean±STD | 36.73±17.72 | 37.19±11.47 |  |
| Min | 23.00 | 18.00 | 0.6633 |
| Max | 94.00 | 58.00 |  |
| Uric Acid level (Mmol/L) Mean±STD | 295.08±139.16 | 289.46±149.62 |  |
| Min | 139.00 | 69.00 | 0.9219 |
| Max | 551.00 | 549.00 |  |
| LDH (U/L) Level Meant±STD | 242.91±117.29 | 306±102.72 |  |
| Min | 1.29 | 128.00 | 0.3152 |
| Max | 389.00 | 410.00 |  |
| CK (U/L) level Meant STD | 86.42±37.05 | 148.36±174.71 |  |
| Min | 50.00 | 15.00 | 0.2169 |
| Max | 149.00 | 737.00 |  |
| AST (U/L) Mean±STD | 34.36±7.7 | 38.93a16.26 |  |
| Min | 23.00 | 22.00 | 0.8721 |
| Max | 53.00 | 77.00 |  |
| ALT (U/L)Mean ± STD | 23.87±10.73 | 31.93±24.92 |  |
| Min | 8.00 | 8.00 | 0.5702 |
| Max | 47.00 | 100.00 |  |
| LDL (mmol/L) Mean±STD | 1.88±0.97 | 1.43±0.61 |  |
| Min | 0.33 | 0.64 | 0.1699 |
| Max | 3.51 | 2.91 |  |
| HDL (mmol/L) Mean±STD | 1.09±0.22 | 1.01±0.36 |  |
| Min | 0.72 | 0.53 | 0.4809 |
| Max | 1.60 | 1.92 |  |
| Triglycerides (mmol/L) Mean±STD | 1.47±1.3 | 1.6±1.49 |  |
| Min | 0.40 | 0.49 | 0.3439 |
| Max | 4.74 | 6.35 |  |

Table S11: Lab results for Visit 5 (12 months ±14 days)

|  | Carglumic acid  N=15 | Standard therapy  N=16 | | P-value | |
| --- | --- | --- | --- | --- | --- |
| PT (Sec)  Up normal n(%) | 11(73.33%) | 1l (78.57%) | | 1.0000 | |
| APTT (sec)  Up normal n(%) | 8(53.33%) | 8(57.14%) | | 1.0000 | |
| INR  Up normal n(%) | 12(80%) | 11(78.57%) | | 1.0000 | |
| Glucose level (mmol/L) Mean±STD | 4.86±0.54 | 5.09±1.27 | |  | |
| Min | 4.20 | 3.30 | | 0.5463 | |
| Max | 6.00 | 7.10 | |  | |
| Urea level (mg/dL) Mean±STD | 5.89±2.36 | 5.47±4.18 | |  | |
| Min | 1.70 | 0.70 | | 0.7327 | |
| Max | 12.00 | 16.00 | |  | |
| Creatinine level (Mmol/L) Mean±STD | 40.8±15.02 | 42.5±7.32 | |  | |
| Min | 13.00 | 31.00 | | 0.6959 | |
| Max | 82.00 | 85.00 | |  | |
| Uric Acid level (Mmol/L) Mean±STD | 268.73±117.51 | 304.14±142.59 | |  | |
| Min | 93.00 | 104.00 | | 0.5130 | |
| Max | 533.00 | 681.00 | |  | |
| LDH (U/L) Level Meant±STD | 306.88±111.25 | 307.29±172.19 | |  | |
| Min | 210.00 | 3.72 | | 0.3329 | |
| Max | 564.00 | 534.00 | |  | |
| CK (U/L) level Mean±STD | 97.27±96.82 | 113.73±70.55 | |  | |
| Min | 17.00 | 9.00 | | 0.2756 | |
| Max | 347.00 | 230.00 | |  | |
| AST (U/L) Mean±STD | 37.57±7.18 | 43.36±20.45 |  | |  |
| Min | 27.00 | 20.00 | 0.7421 | |  |
| Max | 48.00 | 90.00 |  | |  |
| ALT (U/L) Mean±STD | 28.43± l3. 1 l | 25.86±12.44 |  | |  |
| Min | 10.00 | 6.00 | 0.5990 | |  |
| Max | 54.00 | 47.00 |  | |  |
| LDL (mmol/L) Mean±STD | 1.78±0.75 | 1.68±0.69 |  | |  |
| Min | 0.62 | 0.68 | 0.6294 | |  |
| Max | 3.72 | 3.34 |  | |  |
| HDL (mmol/L) Mean±STD | 1.09±0.32 | 1.11±0.38 |  | |  |
| Min | 0.67 | 0.50 | 0.8686 | |  |
| Max | 1.56 | 1.77 |  | |  |
| Triglycerides (mmol/L) Mean±STD | 1.05±0.58 | 1.95±2.36 |  | |  |
| Min | 0.45 | 0.74 | 0.1027 | |  |
| Max | 2.63 | 9.90 |  | |  |

Table S12: Lab results for Visit 6 (18 months ± 14 days)

|  | Carglumic acid  N=14 | Standard therapy  N=16 | P-value |
| --- | --- | --- | --- |
| PT (Sec)  Up normal n(%) | 8(72.73%) | 8(57.14%) | 0.6766 |
| APTT (sec)  Up normal n(%) | 5(45.45%) | 10(71.43%) | 0.2406 |
| INR  Up normal n(%) | 7(63.64%) | 13(92.86%) | 0.1333 |
| Glucose level (mmol/L) Mean±STD | 4.82±0.86 | 5.39±1.34 |  |
| Min | 3.60 | 4.30 | 0.3176 |
| Max | 6.20 | 9.80 |  |
| Urea level (mg/dL) Mean±STD | 5.68±2.41 | 5.58±3.19 |  |
| Min | 1.50 | 0.60 | 0.9265 |
| Max | 10.00 | 12.00 |  |
| Creatinine level (Mmol/L) Mean±STD | 40±26.09 | 40.06±13.61 |  |
| Min | 16.00 | 23.00 | 0.3819 |
| Max | 127.00 | 71.00 |  |
| Uric Acid level (Mmol/L) Mean±STD | 320±140.9 | 327.36±157.58 |  |
| Min | 144.00 | 174.00 | 0.8563 |
| Max | 523.00 | 601.00 |  |
| LDH (U/L) Level Mean±STD | 276±77.44 | 294.86±77.02 |  |
| Min | 196.00 | 206.00 | 0.7723 |
| Max | 389.00 | 438.00 |  |
| CK (U/L) level Mean±STD | 109.36±98.55 | 102±83.1 |  |
| Min | 33.00 | 9.00 | 0.9098 |
| Max | 389.00 | 236.00 |  |
| AST (U/L) Mean±STD | 33.92±12.99 | 41±15.31 |  |
| Min | 21.00 | 24.00 | 0.2034 |
| Max | 69.00 | 72.00 |  |
| ALT (U/L) Mean±STD | 26.85±20.46 | 23.5±11.52 |  |
| Min | 6.00 | 6.00 | 0.9419 |
| Max | 89.00 | 52.00 |  |
| LDL (mmol/L) Mean±STD | 1.91±0.63 | 1.38±0.55 |  |
| Min | 1.08 | 0.76 | 0.0644 |
| Max | 3.14 | 2.26 |  |
| HDL (mmol/L) Mean±STD | 0.97±0.44 | 1.12±0.47 |  |
| Min | 0.37 | 0.50 | 0.4579 |
| Max | 1.66 | 1.91 |  |
| Triglycerides (mmol/L) Mean±STD | 1.41±1.43 | 1.46±0.85 |  |
| Min | 0.33 | 0.63 | 0.3616 |
| Max | 4.66 | 3.06 |  |

Table S13: vital signs as well as the growth parameters

|  | Carglumic acid  N=16 | Standard therapy  N=17 | P-value |
| --- | --- | --- | --- |
| Systolic Blood Pressure Mean±STD | 90.85±23.99 | 96±12.08 | 0.9746 |
| Diastolic Blood Pressure Mean±STD | 58.05±16.91 | 62.13±10.46 | 0.4071 |
| Heart Rate Mean±STD | 109.57±28.55 | l 17.56±l 1.22 | 0.3985 |
| Respiratory Rate Mean±STD | 24.33±11.37 | 25.81±5.0 | 0.1201 |
