# Supplementary material for: Long-term effectiveness of carglumic acid in patients with propionic acidemia (PA) and methylmalonic acidemia (MMA): a randomized clinical trial
Source: Orphanet J Rare Dis. 2021 Oct 11;16:422. doi: 10.1186/s13023-021-02032-8 (PMC8507242; doi:10.1186/s13023-021-02032-8)
Supplement: Supplementary file 2 — Additional file 2. Fig. S1: The Emergency room admissions distribution among the two diseases in the two arms of the study. Fig. S2 The Emergency room admissions distribution between the two arms of the study. Fig. S3 Distribution of ammonia levels over time. The arms showed comparable ammonia levels throughout the study. Fig. S4 Ammonia level distribution between the two groups throughout the study visits. Fig. S5 Kaplan–Meier plot to evaluate the time to first emergency room visit between the study groups. The plot did not show any statistically significant difference. [file 13023_2021_2032_MOESM2_ESM.docx]

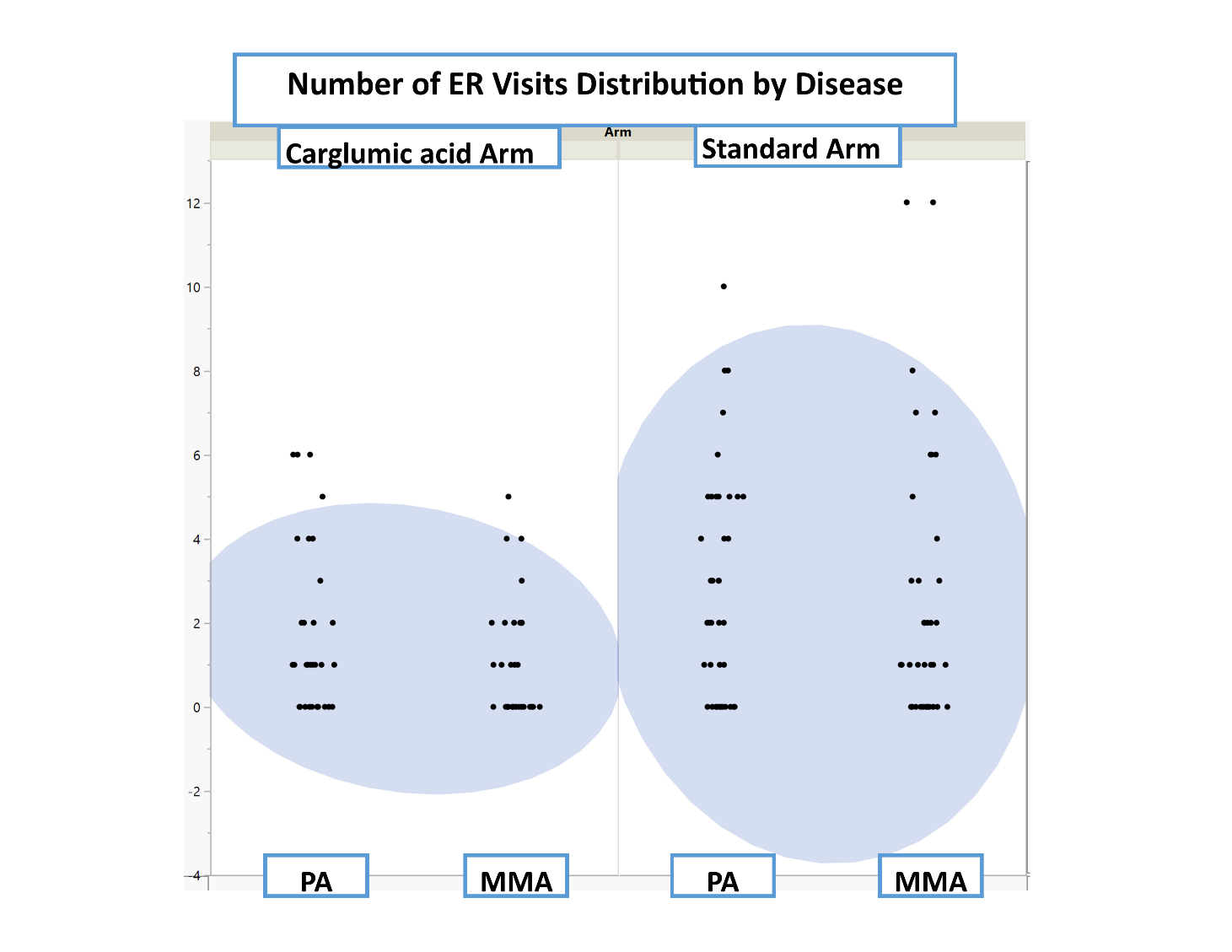


Figure S1: The Emergency room visits distribution among the two diseases in the two arms of the study.


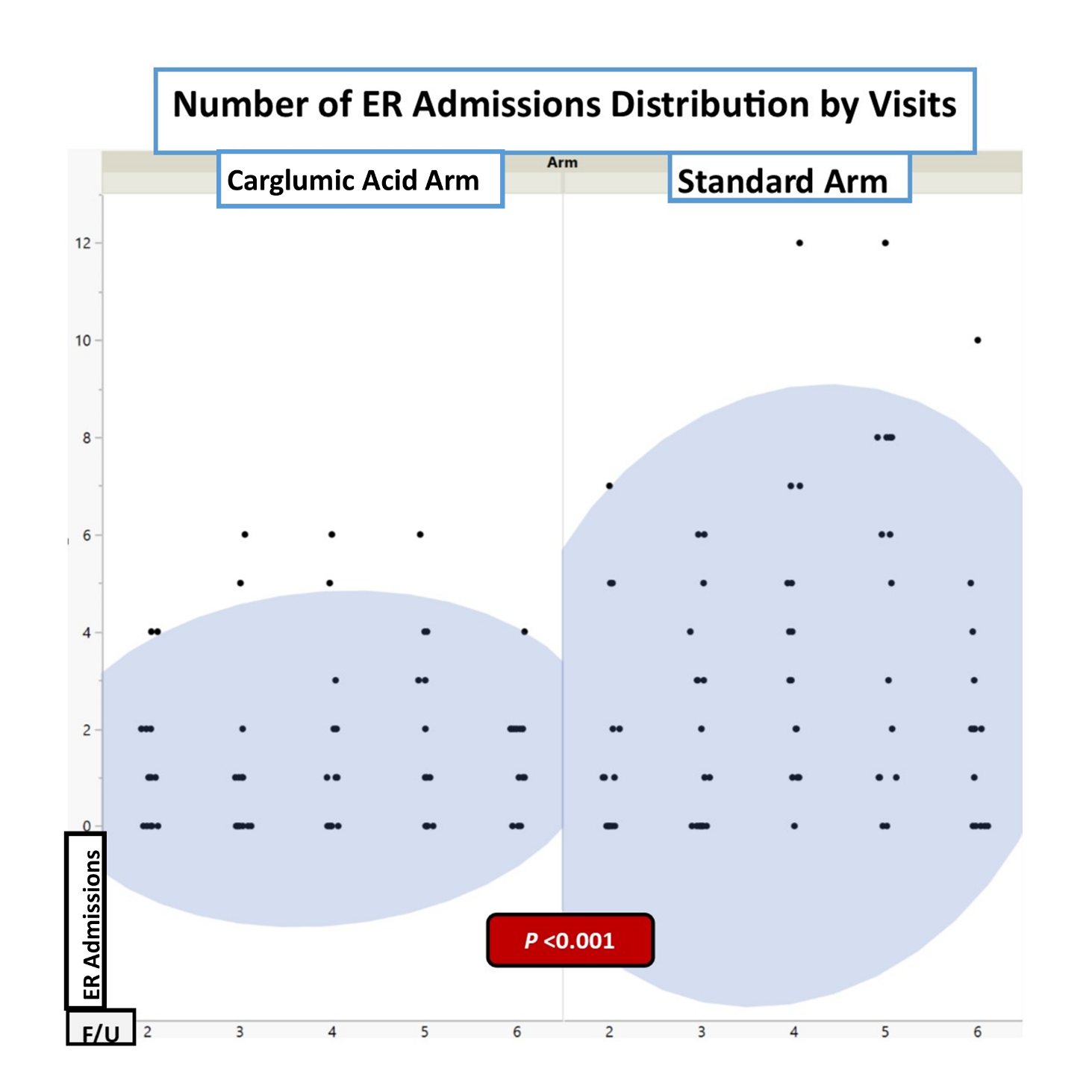


Figure S2: The Emergency room admissions distribution between the two arms of the study.


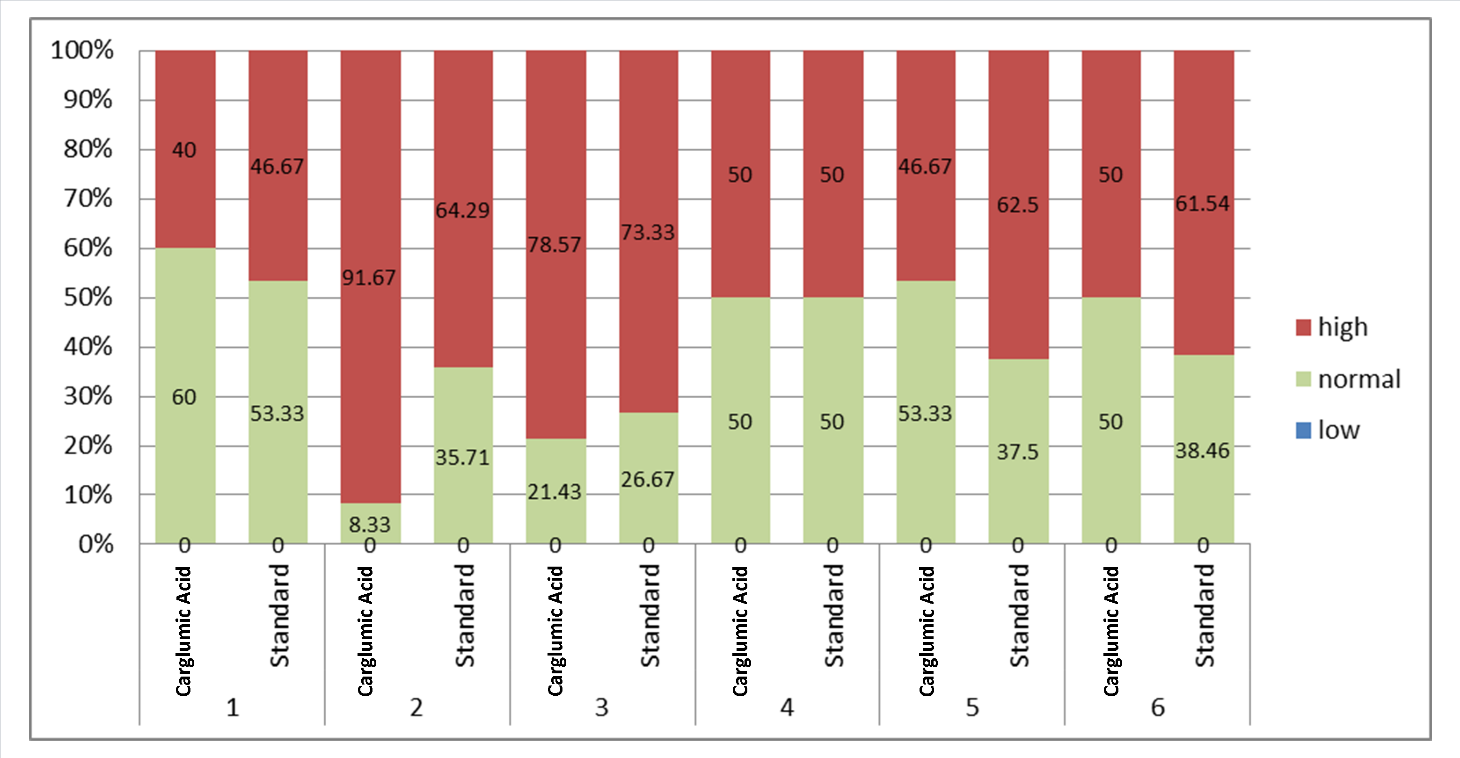


## Figure S3: Distribution of ammonia levels over time. The arms showed comparable ammonia levels throughout the study.


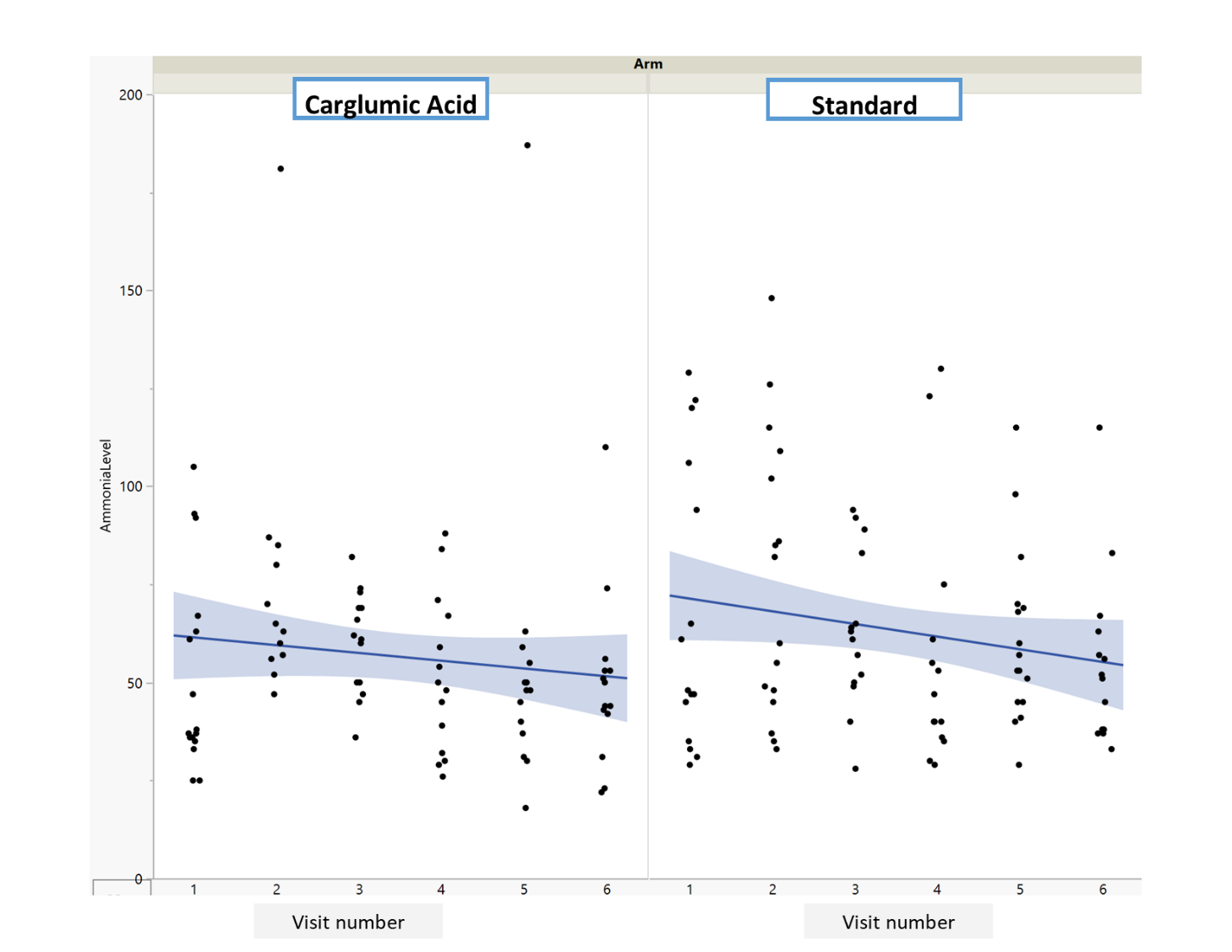


Figure S4: Ammonia level distribution between the two groups throughout the study visits


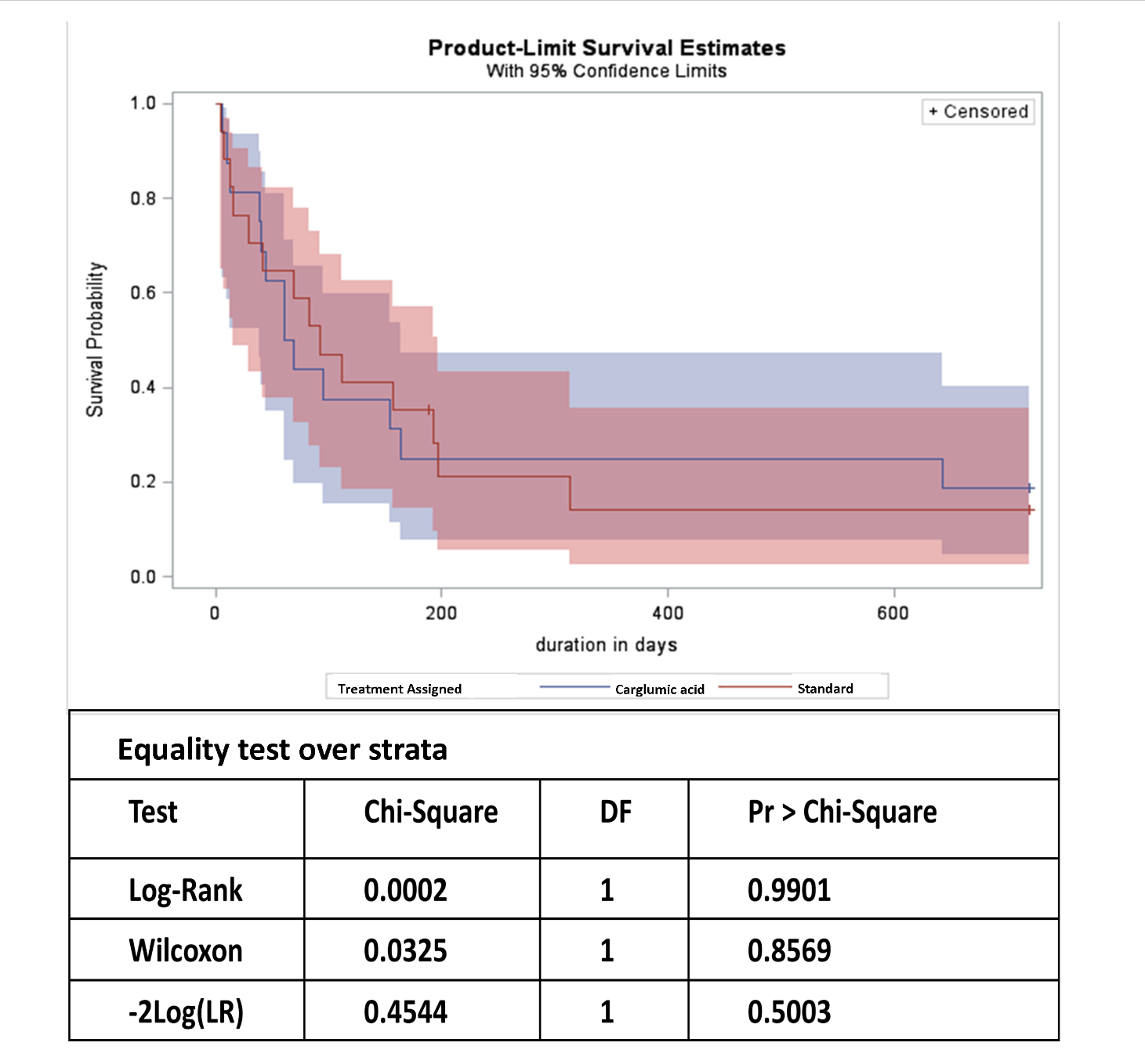


Figure S5: Kaplan-Meier plot to evaluate the time to first emergency room admissions between the study groups. The plot did not show any statistically significant difference.
