# Supplementary material for: Long-term effectiveness of carglumic acid in patients with propionic acidemia (PA) and methylmalonic acidemia (MMA): a randomized clinical trial
Source: Orphanet J Rare Dis. 2021 Oct 11;16:422. doi: 10.1186/s13023-021-02032-8 (PMC8507242; doi:10.1186/s13023-021-02032-8)
Supplement: Supplementary file 3 — Additional file 3. Fig. S6: The average total protein intake per KG per day for the two groups over time. [file 13023_2021_2032_MOESM3_ESM.docx]

This figure depicts the average total protein intake per KG per day for the two groups over time.

| **Model Information** | |
| --- | --- |
| **Data Set** | WORK.NDATA |
| **Dependent Variable** | Total_Proein_KG_DAY |
| **Covariance Structure** | Autoregressive |
| **Subject Effect** | MRN |
| **Estimation Method** | REML |
| **Residual Variance Method** | Profile |
| **Fixed Effects SE Method** | Model-Based |
| **Degrees of Freedom Method** | Between-Within |

| **Class Level Information** | | |
| --- | --- | --- |
| **Class** | **Levels** | **Values** |
| **visit** | 6 | 1 2 3 4 5 6 |
| **ID** | 31 | 1 2 3 4 5 7 8 12 14 15 16 17 18 19 20 21 22 23 25 26 28 30 31 32 34 35 36 37 38 40 41 |
| **Study_arm** | 2 | Carglumic acid Standard |
| **MRN** | 31 | 734066 2120056 2356144 2399252 2427573 2509120 2592919 2646530 2654420 2664765 2669222 2676875 2688279 2701094 2702327 2808322 2810803 427010958 430024561 430044896 432038727 432053251 433049494 433056001 434047598 435013169 435013993 435038128 435044230 437008708 438001919 |

| **Dimensions** | |
| --- | --- |
| **Covariance Parameters** | 2 |
| **Columns in X** | 21 |
| **Columns in Z** | 0 |
| **Subjects** | 31 |
| **Max Obs per Subject** | 6 |

| **Number of Observations** | |
| --- | --- |
| **Number of Observations Read** | 186 |
| **Number of Observations Used** | 186 |
| **Number of Observations Not Used** | 0 |

| **Iteration History** | | | |
| --- | --- | --- | --- |
| **Iteration** | **Evaluations** | **-2 Res Log Like** | **Criterion** |
| **0** | 1 | 262.44502438 |  |
| **1** | 2 | 79.65978661 | 0.00000001 |

| Convergence criteria met. |
| --- |

| **Estimated R Matrix for MRN 734066** | | | | | | |
| --- | --- | --- | --- | --- | --- | --- |
| **Row** | **Col1** | **Col2** | **Col3** | **Col4** | **Col5** | **Col6** |
| **1** | 0.2199 | 0.1864 | 0.1579 | 0.1338 | 0.1134 | 0.09605 |
| **2** | 0.1864 | 0.2199 | 0.1864 | 0.1579 | 0.1338 | 0.1134 |
| **3** | 0.1579 | 0.1864 | 0.2199 | 0.1864 | 0.1579 | 0.1338 |
| **4** | 0.1338 | 0.1579 | 0.1864 | 0.2199 | 0.1864 | 0.1579 |
| **5** | 0.1134 | 0.1338 | 0.1579 | 0.1864 | 0.2199 | 0.1864 |
| **6** | 0.09605 | 0.1134 | 0.1338 | 0.1579 | 0.1864 | 0.2199 |

| **Estimated R Correlation Matrix for MRN 734066** | | | | | | |
| --- | --- | --- | --- | --- | --- | --- |
| **Row** | **Col1** | **Col2** | **Col3** | **Col4** | **Col5** | **Col6** |
| **1** | 1.0000 | 0.8473 | 0.7179 | 0.6083 | 0.5154 | 0.4367 |
| **2** | 0.8473 | 1.0000 | 0.8473 | 0.7179 | 0.6083 | 0.5154 |
| **3** | 0.7179 | 0.8473 | 1.0000 | 0.8473 | 0.7179 | 0.6083 |
| **4** | 0.6083 | 0.7179 | 0.8473 | 1.0000 | 0.8473 | 0.7179 |
| **5** | 0.5154 | 0.6083 | 0.7179 | 0.8473 | 1.0000 | 0.8473 |
| **6** | 0.4367 | 0.5154 | 0.6083 | 0.7179 | 0.8473 | 1.0000 |

| **Covariance Parameter Estimates** | | |
| --- | --- | --- |
| **Cov Parm** | **Subject** | **Estimate** |
| **AR(1)** | MRN | 0.8473 |
| **Residual** |  | 0.2199 |

| **Fit Statistics** | |
| --- | --- |
| **-2 Res Log Likelihood** | 79.7 |
| **AIC (Smaller is Better)** | 83.7 |
| **AICC (Smaller is Better)** | 83.7 |
| **BIC (Smaller is Better)** | 86.5 |

| **Null Model Likelihood Ratio Test** | | |
| --- | --- | --- |
| **DF** | **Chi-Square** | **Pr > ChiSq** |
| 1 | 182.79 | <.0001 |

| **Solution for Fixed Effects** | | | | | | | |
| --- | --- | --- | --- | --- | --- | --- | --- |
| **Effect** | **Study arm** | **visit** | **Estimate** | **Standard Error** | **DF** | **t Value** | **Pr > \|t\|** |
| **Intercept** |  |  | 1.9125 | 0.1172 | 29 | 16.31 | <.0001 |
| **visit** |  | 1 | 0.3194 | 0.1244 | 145 | 2.57 | 0.0113 |
| **visit** |  | 2 | 0.2137 | 0.1154 | 145 | 1.85 | 0.0661 |
| **visit** |  | 3 | 0.2944 | 0.1038 | 145 | 2.84 | 0.0052 |
| **visit** |  | 4 | 0.1350 | 0.08806 | 145 | 1.53 | 0.1275 |
| **visit** |  | 5 | 0.08937 | 0.06479 | 145 | 1.38 | 0.1699 |
| **visit** |  | 6 | 0 | . | . | . | . |
| **Study_arm** | Carglumic acid |  | 0.2542 | 0.1685 | 29 | 1.51 | 0.1424 |
| **Study_arm** | Standard |  | 0 | . | . | . | . |
| **visit*Study_arm** | Carglumic acid | 1 | 0.08463 | 0.1789 | 145 | 0.47 | 0.6369 |
| **visit*Study_arm** | Standard | 1 | 0 | . | . | . | . |
| **visit*Study_arm** | Carglumic acid | 2 | 0.1376 | 0.1659 | 145 | 0.83 | 0.4084 |
| **visit*Study_arm** | Standard | 2 | 0 | . | . | . | . |
| **visit*Study_arm** | Carglumic acid | 3 | -0.03237 | 0.1492 | 145 | -0.22 | 0.8285 |
| **visit*Study_arm** | Standard | 3 | 0 | . | . | . | . |
| **visit*Study_arm** | Carglumic acid | 4 | -0.00833 | 0.1266 | 145 | -0.07 | 0.9476 |
| **visit*Study_arm** | Standard | 4 | 0 | . | . | . | . |
| **visit*Study_arm** | Carglumic acid | 5 | -0.01271 | 0.09314 | 145 | -0.14 | 0.8917 |
| **visit*Study_arm** | Standard | 5 | 0 | . | . | . | . |
| **visit*Study_arm** | Carglumic acid | 6 | 0 | . | . | . | . |
| **visit*Study_arm** | Standard | 6 | 0 | . | . | . | . |

| **Type 3 Tests of Fixed Effects** | | | | |
| --- | --- | --- | --- | --- |
| **Effect** | **Num DF** | **Den DF** | **F Value** | **Pr > F** |
| **visit** | 5 | 145 | 4.22 | 0.0013 |
| **Study_arm** | 1 | 29 | 3.77 | 0.0620 |
| **visit*Study_arm** | 5 | 145 | 0.71 | 0.6158 |

We modeled the Total Protein per KG/DAY for the two groups using a linear mixed model for the longitudinal data. The main effects of the model are the time as the number of visits, and the study groups ( Carglumic acid and Standard). There was a significant decrease in the Total Protein per KG/DAY over time (p=0.0013). However, there was no significant difference between the two treatment groups in the Total Protein per KG/DAY (p=0.062). There was no significant difference between the two treatment groups over time as in the interaction effect (p=0.6158).
